# Lifespan brain age prediction based on multiple EEG oscillatory features and sparse group lasso

**DOI:** 10.3389/fnagi.2025.1559067

**Published:** 2025-07-22

**Authors:** Shiang Hu, Xue Xiang, Xiaolong Huang, Yan Lu, Xulai Zhang, Dezhong Yao, Pedro A. Valdes-Sosa

**Affiliations:** ^1^Anhui Provincial Key Lab of Multimodal Cognitive Computation, Key Lab of Intelligent Computing and Signal Processing of Ministry of Education, School of Computer Science and Technology, Anhui University, Hefei, China; ^2^Department of Applied Statistics, Stony Brook Institute at Anhui University, Hefei, China; ^3^Affiliated Psychological Hospital of Anhui Medical University, Anhui Mental Health Center, Hefei Fourth People's Hospital, Hefei, China; ^4^MOE Key Lab for Neuroinformation, School of Life Science and Technology, University of Electronic Science and Technology of China, Chengdu, China

**Keywords:** EEG, neural oscillation, brain age, sparse group LASSO, layerwise relevance propagation

## Abstract

**Introduction:**

The neural dynamics underlying cognition and behavior change greatly during the lifespan of brain development and aging. EEG is a promising modality due to its high temporal resolution in capturing neural oscillations. Precise prediction of brain age (BA) based on EEG is crucial to screening high-risk individuals from large cohorts. However, the lifespan representation of the EEG oscillatory features (OSFs) is largely unclear, limiting practical BA applications in clinical scenarios. This study aims to build an interpretable BA prediction model through prior knowledge and sparse group lasso.

**Methods:**

Based on the multinational cross-spectral (MNCS) dataset that covers 5–97 years, (1) we extracted four groups of OSFs, such as aperiodic parameters, periodic parameters, power-ratio, and relative power; (2) the OSFs trajectories evolving with age and the OSF importance topographies were mapped using the generalized additive model for location, scale and shape (GAMLSS) and Pearson's correlation coefficient (PCC); (3) the inter-oscillatory dependency coefficients (ODCs) were extracted by the sparse group lasso; (4) the fusion of OSFs and ODCs was flattened and fed into a three-layer fully connected neural network (FCNN); the FCNN interpretability was analyzed by Layerwise Relevance Propagation and 10-fold cross-validation.

**Results:**

The results showed that the FCNN model that incorporated ODC significantly improved the prediction of BA (MAE = 2.95 years, *R*^2^ = 0.86) compared to the use of only OSF (MAE = 3.44 years, *R*^2^ = 0.84).

**Discussion:**

In general, this study proposed a BA prediction model named NEOBA by systematically employing OSFs and highlighting the interpretability of the model, which holds broad promise by integrating normative modeling for precise individual stratification.

## 1 Introduction

The brain aging process is a multidimensional phenomenon that involves the omics change at the molecular level and structural and functional changes at the neuronal level (Rutledge et al., 2022; Bethlehem et al., 2022). Age-dependent brain signature trajectories have been the research interests of neuroscientists for decades (Giedd et al., 1999; Jernigan et al., 2001). Magnetic resonance imaging (MRI) has been widely used to model brain age (BA) prediction based on anatomical changes (Franke et al., 2010; Vergun et al., 2013; Cole and Franke, 2017; Amoroso et al., 2019; Mouches et al., 2022; Poloni and Ferrari, 2022). The application of MRI-based BA prediction is limited by the high cost of the MRI scanner, especially in low-middle-income countries and underdeveloped areas. MRI-based BA prediction models become even inefficient in the early stage of mental disorders when structural abnormalities are undetectable. In contrast, electroencephalography (EEG) can effectively capture the neural oscillatory dynamics driven by the excitability of neuronal populations due to its high temporal resolution and non-invasiveness (Cohen, 2017). Neural oscillations are associated with psychophysiological processes such as perception, cognition, motor, and emotional function. From the macroscale view, neural oscillations provide a window to study functional changes in neurodevelopment, maturation, and aging throughout the human lifespan (Siegel et al., 2012; Ward, 2003). The latest quantitative EEG studies highlight the parameterization of neural oscillations by first decomposing the power spectra into aperiodic and periodic/rhythmic components (Donoghue et al., 2020a; Hu et al., 2024), and then quantifying the oscillatory features (OSFs). Common OSFs are aperiodic parameters (AP) that contain offset and exponent, periodic parameters (PP) that include center frequency, power, and bandwidth, and traditional descriptors such as band-limited absolute power (BLAP), power ratios (PR), and relative power (RP).

Existing studies on normative OSFs have drawn tedious conclusions from different age groups. Marshall et al. (2002), Clarke et al. (2001), and Miskovic et al. (2015) demonstrated that PP, RP, and PR in all frequency bands vary with age. Cragg et al. (2011) examined BLAP maturation in early adolescence and observed that BLAP−δ and BLAP−θ decreased with age, accompanied by increases in RP−α2 and RP−β, which was also reported by Hashemi et al. (2016) and Hu et al. (2019a). Smith (2023) found that α peak frequency decreased with age in middle-aged and elderly individuals. Changes in age-related OSFs reflect various functional processes in neurodevelopment and aging (De Bellis, 2001; Larsen and Luna, 2018). A critical point in studying OSFs is whether AP is excluded from the raw power spectral curves (Donoghue et al., 2020a). Previous studies treated BLAP as a direct reflection of oscillatory activity, without considering AP (Schaworonkow and Voytek, 2021). When oscillatory bursts of PP are not present, AP dominates. Even when PP is absent, spectral analysis will show power in the broad frequency band completely driven by AP (Schaworonkow and Voytek, 2021). Therefore, it is impossible to determine whether PP bursts, AP changes, or a combination of both causes BLAP without separating AP from raw spectra.

Existing research suggests that AP changes over the full lifespan. In adults, several studies have reported that the AP-exponent decreases with age (Merkin et al., 2023; Tran et al., 2020; Voytek et al., 2015; Dave et al., 2018). Age-related AP changes greatly from childhood to adolescence. A longitudinal EEG study on 38–203 days of infants showed that the AP-exponent decreases with age (Schaworonkow and Voytek, 2021). An EEG study in children and young adults aged 5–21 years reported flattening of the AP-exponent and a decrease in the AP-offset with age (Tröndle et al., 2023; Cellier et al., 2021). Recently, a similar trend was reported in children and adults aged 3–24 years. Given the emerging evidence, the AP-exponent shows strong variations in aging up to 70 years (Voytek et al., 2015) and childhood aged 4–12 years. Therefore, integrating AP and PP can help accurately assess functional brain change and predict age, with implications for understanding brain development patterns, preventing neural degenerative diseases, and evaluating therapeutic efficacy.

Regarding the prediction of BA, Dimitriadis and Salis (2017) observed reproducible patterns of accelerated brain aging in different frequency bands in resting EEG, highlighting the importance of intrinsic neural oscillations. They used a support vector regressor to develop a linear model based on spatio-temporal EEG features to predict BA. This model was applied to 194 resting EEG recordings of 19–67-year-old adults using a single integrated dynamic functional connectivity graph. The prediction process was conducted under both eyes-open (*R*^2^ = 0.6) and eyes-closed (*R*^2^ = 0.48) conditions. Al Zoubi et al. (2018) used five sets of EEG features across channels and frequency bands and stacked ensemble learning to predict the BA of 468 participants with a mean age of 34.3 years. Their results showed a mean absolute error (MAE) of 6.87 years and a *R*^2^ of 0.37. Vandenbosch et al. (2019) used power features for random forest, support vector machine, and relevance vector machine to predict the BA of 702 juveniles aged 5–18 years. The results showed MAE of 1.22 and 1.46 years, with *R*^2^ of 0.547 and 0.448. Khayretdinova et al. (2022) used data augmentation and deep convolutional neural networks to predict the BA of 1,274 subjects aged 5–88 years using resting-state EEG and obtained the MAE of 5.96 years.

Current EEG research primarily focuses on applying power spectra or spatiotemporal features to predict BA. These researchers often overlook the importance of AP for age prediction. Traditional EEG feature extraction methods mainly concentrate on univariate spectra or multivariate functional connectivity patterns, lacking sufficient recognition of the dependency coefficients between OSFs. Moreover, feature processing and model construction in deep learning are complex and require substantial computational resources, making it difficult to apply in clinical settings, and suffer from poor interpretability. Therefore, developing a more effective BA prediction framework remains a challenge.

The sparse group lasso was commonly used for feature selection (Zhao et al., 2015; Huo et al., 2020; Zhou and Zhu, 2010; Rao et al., 2015; Xu et al., 2023; Scardapane et al., 2017). Specifically, the sparse group lasso imposes an L2 norm constraint on each feature group to ensure that features within the entire group are either all selected or all excluded. Concurrently, it applies an L1 norm constraint on individual features to achieve further sparsification (Simon et al., 2013; Liu et al., 2009). This design enables group coefficients to retain significant feature groups while eliminating irrelevant features, thus improving the generalization capability and predictive accuracy of the model (Zhao et al., 2015; Xu et al., 2023; Huo et al., 2020). However, these studies often focus on the relationship between the selected features and the output, overlooking the relationships within the features themselves. This work attempts to explore a new feature analysis method using the sparse group lasso. This method aims to transcend the binary decision of either “select” or “not select,” instead delving deeper into uncovering the complex connections between features, the inter-OSF dependency coefficients (ODC). The ODC provides richer information for predictive models. These relationships may have previously been underappreciated but have significant implications for understanding neurodynamic changes during brain development and aging processes.

Deep learning models are often considered “black boxes” because of complex non-linear transformations that make it difficult to directly understand the input-output relationship. To enhance the interpretability of neural networks, researchers have introduced attention mechanisms into Recurrent Neural Networks and Convolutional Neural Networks (Mnih et al., 2014; Chorowski et al., 2015; Xu, 2015). These mechanisms dynamically learn and assign attention weights, effectively highlighting which features are most important for a given task. However, this approach increases model complexity and computational requirements. To address interpretability without altering the original network architecture, the Layerwise Relevance Propagation (LRP) algorithm has been proposed as a postprocessing method (Bach et al., 2015; Samek et al., 2016). The LRP process involves backpropagating the network output to the input layer and assigning a relevance score to each input. This process reveals the contribution of each input feature to the final decision. In the field of bioinformatics, LRP has been applied to explain clinical decisions made by deep neural networks (Yang et al., 2018; Böhle et al., 2019). For example, LRP has been used to visualize the CNN decision-making process for Alzheimer's disease (Böhle et al., 2019). Heatmaps are generated to highlight input features that positively impact classification. This visualization helps to understand which parts of the input data are most influential in the network's decisions. In addition, Dobrescu et al. (2019) used LRP to determine the edges of the leaves as a key to the leaf counting task. Li et al. (2022a) developed a spatiotemporal LRP method, which can quantitatively evaluate the temporal and spatial correlations between multiple inputs and predicted energy consumption and provide in-depth explanations based on expert knowledge. Generally, LRP is a method to evaluate the impact of specific image pixels or regions on the prediction by a classifier or regressive models (Dobrescu et al., 2019; Binder et al., 2016). We hope to combine LRP with fully connected layers to analyze the direct impact of OSFs on prediction results and explore which features have the greatest influence, as well as whether these features can be consistent with existing prior knowledge, especially considering the significance of certain features changing with age.

In this study, we propose an interpretable model to predict BA based on prior knowledge and sparse group lasso. The main contributions are: (1) Exploration of age-related OSFs: we integrate AP and investigate the OSFs trajectories across the lifespan based on GAMLSS; (2) Calculation and validation of ODC: we propose ODC based on the sparse group lasso. Our empirical analysis shows that ODC significantly improves the accuracy of BA prediction using resting EEG. This contribution addresses a critical gap in existing methodologies and offers more robust information in the context of EEG BA prediction; (3) Enhancing model interpretability with LRP: we employ LRP to improve the interpretability of the prediction model. The structure of this paper is organized as follows. Section 2 introduces data and model construction methods. Section 3 presents the main results. Detailed discussion is given in Section 4 with the concluding remarks presented in Section 5.

## 2 Methods

### 2.1 MNCS dataset

The multinational EEG cross-spectra dataset (MNCS) (Li et al., 2022b) is currently the largest repository dedicated to EEG spectral profiles of healthy subjects covering the human lifespan. The MNCS encompasses EEG cross-spectra from 12 amplifiers, 14 studies, and 1,966 subjects aged 5–97 years. It was collected from voluntary contributions from nine countries with the support of the International Collaboration Framework Global Brain Consortium (GBC). All subjects provided their informed written consent, allowing the EEG data to be shared for scientific research. All data have been anonymized for privacy protection.

**Figure 1:**
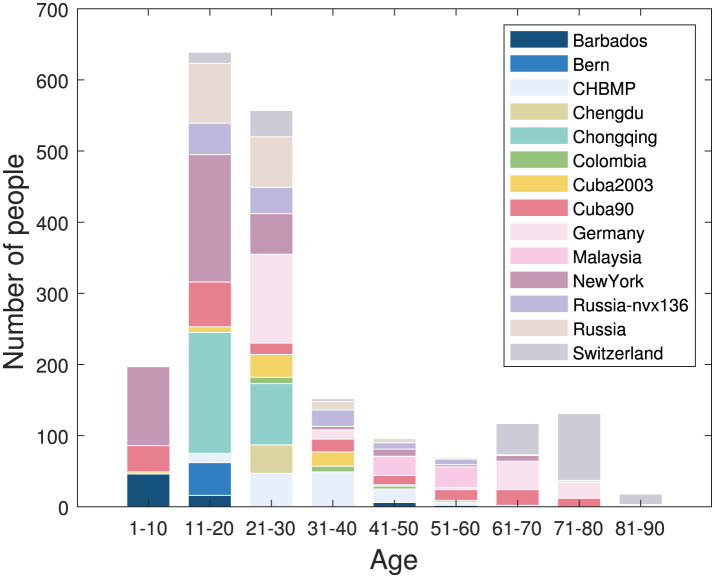
Age distribution of MNCS dataset.

The gender distribution of the sample is nearly even, and the age distribution of 5–97 years is illustrated in Figure 1. Raw EEG data were recorded using 19 channels based on the 10–20 international electrode placement system, including Fp1, Fp2, F3, F4, C3, C4, P3, P4, O1, O2, F7, F8, T3/T7, T4/T8, T5/P7, T6/P8, Fz, Cz, Pz. Data shared from individual sites include EEG cross-spectra, demographic information such as age, gender, and country, and equipment information, including amplifiers, reference electrodes, and experiment details. The cross spectra from each site were computed using a unified script datagatherer. Li et al. (2022b), which sets the Bartlett method (Møller, 1986), the averaged non-overlapping periodograms and the frequency range of 1.17–19.14 Hz with a 0.39 Hz interval. The window size to calculate the cross-spectra was 2.56 s. The noise from the power line was removed at the main AC frequency of the respective country. The upper frequency was limited to accommodate legacy EEG cross spectra of 211 subjects aged 5–80 years (Bosch-Bayard et al., 2020), for which the raw EEG time series were not saved due to sampling and memory limitations of the original amplifers.

The preprocessing of the MNCS dataset included: (1) extracting diagonals from cross spectra as power spectra; (2) applying average reference to standardize the scalp EEG reference and discarding the electrode Pz to reduce the electrode from 19 to 18 because the average reference has the property of rank deficiency by 1 (Hu et al., 2019b, 2018c; Yao et al., 2019); (3) correcting the global scale factor from the raw power spectra; (4) taking the log transformation for all power spectra; (5) performing multisite harmonization by the general linear additive model and the Nadaraya–Watson kernel regression considering age and frequency as fixed effects and gender and batch effects as random effects to minimize the batch effects of inconsistent devices, studies, and counties of multi-site recording (Li et al., 2022b). The preprocessing and harmonization of the MNCS finally involved the 1,966 subjects of multinational power spectra with 18 electrodes and 47 frequency bins, a 1,966 × 18 × 47 tensor.

### 2.2 Data quality control

It is vital to ensure that the data set contains only healthy participants with the assumption of brain age = chronological age, which is the basis for the study of brain age prediction. The details of the inclusion/exclusion criteria, the notes before EEG recording, the instructions during EEG recording, and the EEG preprocessing after EEG recording were given in the Supplementary Appendix “Cohort description.xlsx” with respective references. As indicated in (Li et al., 2022b), (1) these criteria are sufficient and equally stringent to guarantee a sample of functionally healthy subjects; (2) To be accepted into the MNCS study, the shared data had to be part of a normative study or a control group with explicit inclusion/exclusion criteria; (3) the MNCS data were obtained from ≥ 1 min artifact-free, eyes closed, quasistationary, resting EEG. Detailed examples of inclusion/exclusion criteria are provided in Hernandez-Gonzalez et al. (2011) and Bosch-Bayard et al. (2020). The individual study who contributed data to MNCS was required to strictly adhere to the inclusion criteria as Valdes-Sosa et al. (2021). The EEG was usually recorded in the morning after breakfast and before lunch. The duration of the recordings varied from 5 min to half an hour. Subjects were instructed to remain awake and eyes closed during the recording. At least two trained and certified clinical neurophysiologists ensured that subjects remained awake by both observation of behavior and inspection of online EEG recordings.

**Figure 2:**
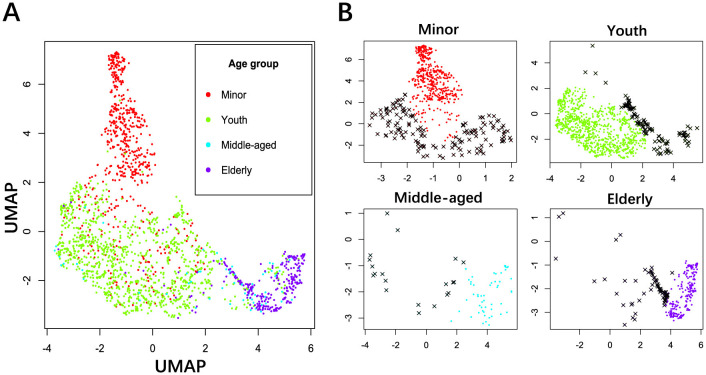
Outlier detection of power spectra using UMAP. **(A)** Power distribution in a 2D space for all age groups in different colors. **(B)** Outlier detection for the age groups of minors, youth, middle age, and elderly. The threshold was set to 0.975 to identify extreme outliers. Black crosses: outliers, solid dots: acceptable samples.

Both normative modeling and BA prediction are to solve the regression problem, which is a machine learning task. Appropriate removal of outliers helps prevent overfitting while enhancing generalizability and robustness. The MNCS age values were divided into groups according to WHO age brackets, such as the minors (≤18 years), youth (19–45 years), middle age (46–60 years), and elderly (>60 years). The uniform manifold approximation and projection (UMAP) (McInnes et al., 2018) is a nonlinear algorithm to map high-dimensional data to a low-dimensional space while preserving the local and global structure of the data points. The *z*-score transformation was applied to the power spectra to detect outliers better using UMAP. Taking all power spectra across the electrodes and the frequencies as features and subjects as samples, UMAP reduced the dimensionality from 1,966 × 18 × 47 to 1,966 × 2, as shown in Figure 2. Then, the robust distance (Rousseeuw and Leroy, 1987) between each subject within each group and the clustering centroid of the age group was calculated using the minimum covariance determinant algorithm. Finally, a threshold of 0.975 was established to identify outliers, resulting in 338 subjects being recognized as outliers. Note that the identical threshold was applied as well as (Li et al., 2022b). One should be cautious when removing outliers because the large variation may easily present in the rapid transition stages such as from puberty to adults and from adults to the elderly. Although removal of outliers can help for normative modeling, we should avoid removing important physiological changes. The trivial step in EEG preprocessing can accumulate substantial impacts on postprocessing outcomes (Hu et al., 2025; Delorme, 2023).

### 2.3 Extraction of oscillatory features (OSFs)

Four groups of neural OSFs were extracted, which are the aperiodic parameters (AP) consisting of exponent and offset, the periodic parameters (PP) including center frequency, power and bandwidth, the power ratio (PR) of two frequency bands such as θ/β, δ/θ, δ/α, θ/α, the relative power (RP) in the frequency bands δ (1–4 Hz), θ (4–8 Hz), α (8–12 Hz), and β (12–20 Hz).

#### 2.3.1 Separating aperiodic and periodic components

Neural oscillations that appear as EEG wave traces are intrinsically the superposition of the aperiodic component and the periodic component. FOOOF (Donoghue et al., 2020b) toolbox can simultaneously perform spectral decomposition and estimate the parameters of the aperiodic and periodic components. FOOOF assumed the additive model in the log space as:


(1) $$\begin{array}{l} PSD=L+\overset{n}{\underset{n=1}{\sum }}G_{n} \end{array}$$

where *PSD* is the log power, *L* is the aperiodic component, and *G*_*n*_ is the periodic component.

#### 2.3.2 Aperiodic parameters (AP)

The shape property of the aperiodic component is decreasing monotonically. It is fitted as a power-law function throughout the frequency range, indicating that the background aperiodic spectra decay exponentially with frequency. The APs are the aperiodic exponent (slope) and offset (Donoghue et al., 2020b) that are expressed in the power-law function as:


(2) $$\begin{array}{l} L\left(F\right)=b-log\left(k+F^{\chi }\right) \end{array}$$

where *F* is a vector of frequencies, *b* is the offset, *k* is the optional knee parameter, χ is the exponent. The exponent χ reflects the steepness of the power spectral decay across frequencies, while the offset *b* reflects the broadband displacement of power across frequencies.

#### 2.3.3 Periodic parameters (PP)

The shape property of the periodic component is the unimodality, which forms a prominent spectral peak in the spectral curve, first increasing and then decreasing, both of which follow the monotonicity. Each periodic component *G*_*n*_ in the form of a spectral peak is individually modeled using a Gaussian kernel function as:


(3) $$\begin{array}{l} G_{n}\left(F\right)=p_{n}*exp\left[\frac{-{\left(F-c_{n}\right)}^{2}}{2w_{n}^{2}}\right] \end{array}$$

where *p*_*n*_ is the power, *c*_*n*_ is the center frequency (Hz), and *w*_*n*_ is the bandwidth.

#### 2.3.4 Power ratio (PR) between two frequency bands

With the multinational power spectra, it is empirically partitioned into δ, θ, α, and low β frequency bands with 1–4, 4–8, 8–12, 12–20 Hz, respectively. The absolute power for each specific frequency band is the sum of power spectral values at bandpass frequencies. The power ratio(PR) is the ratio between the absolute powers of two frequency bands that are of interest, which is expressed as:


(4) $$\begin{array}{l} PR_{fb_{1}/fb_{2}}=\frac{\sum_{lc_{1}}^{hc_{1}}PSD\left(fb_{1}\right)}{\sum_{lc_{2}}^{hc_{2}}PSD\left(fb_{2}\right)} \end{array}$$

where the *fb* refers to the frequency band passing through the low-cut off frequency *lc* and high-cut off frequency *hc*. In this study, the four PRs calculated were PR−δ/θ, PR−δ/α, PR−θ/β, and PR−θ/α.

#### 2.3.5 Relative power (RP)

Similar to PR, relative power (RP) is a measure to eliminate the absolute power difference between individuals. The RP is expressed as


(5) $$\begin{array}{l} RP_{fb}=\frac{\sum_{lc}^{hc}PSD\left(fb\right)}{\sum PSD\left(F\right)} \end{array}$$

where *F* is a vector of all frequencies within 1.17–19.14 Hz, and the denominator is the sum of absolute powers from all frequency bands. Here, the four calculated RPs are RP−δ, RP−θ, RP−α, and RP−β.

### 2.4 Age-dependent trajectory of OSFs

The generalized additive model for location, scale, and shape (GAMLSS) is a semiparametric regression method that allows us to model the entire distribution of a response variable (Stasinopoulos and Rigby, 2008). In this study, GAMLSS was applied to map the trajectories of features that change with age. For each feature, we first calculated the mean value for each electrode, then constructed the GAMLSS model using cubic spline smoothing and the Box-Cox-t family of distributions (Rigby and Stasinopoulos, 2006). We modeled the evolutionary trajectories of 13 features from four groups. In addition, we set the 25%, 50%, and 75% quantiles to better understand the evolutionary trajectories of OSFs.

### 2.5 Electrode-wise correlation of OSFs with BA

For each subject, 13 OSFs of four groups were extracted from 18 electrodes for the prediction of BA. The Pearson correlation coefficient (PCC) (Lee Rodgers and Nicewander, 1988) was applied to calculate the relationship between each OSF on each electrode and BA. This helps to determine which features are significantly correlated with BA prediction, and PCC quantifies the contribution of each feature to predicting BA. After a detailed analysis of the correlation between each OSF per electrode and age, we divided the 18 electrodes into five scalp regions according to the electrode locations. Each region contains a specific set of electrodes: frontal (Fp1, Fp2, F7, F8, F3, F4, Fz), central (C3, C4, Cz), parietal (P3, P4), occipital (O1, O2), and temporal (T3, T4, T5, T6). This division allows for further exploration of the importance of OSFs in brain regions and understanding of differences among brain regions in age prediction. The PCCs of each brain region were the mean of the PCCs of the electrodes located in that brain region.

### 2.6 Inter-OSFs dependency coefficients (ODC)

Because bivariate pairwise correlations between features can ignore the potential effects of other features, the sparse group lasso (Liu et al., 2009) is used to quantify the relationships among OSFs. The OSFs of each subject are represented as $\text{X}=\left[{\text{x}}_{1},...,{\text{x}}_{m}\right]\in R^{d\times m}$, with a total of *m* features, where ${\text{x}}_{m}={\left[e_{1},e_{2},e_{3},...,e_{d}\right]}^{T}\in R^{d}$ represents the *m*-th OSF extracted from all electrodes. Each **x**_*i*_ is considered a target vector that can be fitted by a linear combination of other *m* − 1 features, that is, regressors.

The sparse regression model is defined as **x**_*i*_ = **Aw**, where **w** is the regression coefficient. In the *i*-th regression, the data matrix **A** = [**x**_1_, **x**_2_, ...**x**_*i*−1_, **x**_*i* + 1_, ...**x**_*m*_] contains all feature vectors except for **x**_*i*_. Sparse solutions are achieved by solving the Moreau-Yosida regularization (Liu and Ye, 2010) as


(6) $$\begin{array}{l} \underset{\text{w}}{min}\frac{1}{2}∥{\text{x}}_{i}-\text{A}\text{w}{∥}_{2}^{2}+\lambda_{1}∥\text{w}{∥}_{1}+\lambda_{2}\overset{g}{\underset{j=1}{\sum }}z_{j}^{g}∥{\text{w}}_{G_{j}}{∥}_{2} \end{array}$$

where *A* ∈ *R*^*d* × (*m* − 1)^, ${\text{x}}_{i}\in R^{d}$, **w** ∈ *R*^*m*−1^ are divided into *g* non-overlapping groups **w**_*G*_*j*__ (*j* = 1, ..., *g*). $z_{j}^{g}$ represents the weight for the *j*-th group as $z_{j}^{g}=\overset{n_{j}}{\underset{i=1}{\sum }}|PCC\left({\text{x}}_{i},{\text{a}}_{i}^{n_{j}}\right)|$ with ${\text{a}}_{i}^{n_{j}}\neq {\text{x}}_{i}$ and *PCC* indicating the Pearson correlation coefficient between **x**_*i*_ and the other *n*_*j*_ feature vectors in the *j*-th group. Note that if ${\text{a}}_{i}^{n_{j}}={\text{x}}_{i}$, ${\text{a}}_{i}^{n_{j}}$ should be excluded as the PCC with **x**_*i*_ itself is one. $z_{j}^{g}$ reflects the general similarity between the target OSF and a specific group of OSFs. The sparsity between groups is controlled by the product of λ_2_ and the weight $z_{j}^{g}$ of the respective group. This aggregation method helps us to better capture the dependencies and interactions between features within a group, which is crucial for sparse group lasso models to identify important feature groups and their contributions to regression.

The optimal (λ_1_, λ_2_) was selected when the least MAE was obtained using the grid search and cross-validation approach. The SLEP MATLAB package (Liu et al., 2009) was used for optimization. The λ ranges were set in $\lambda_{1}\in \left\{2^{-1},2^{-2},2^{-3},...2^{-10}\right\},\lambda_{2}\in \left\{0,0.1,0.2,0.3,...,0.9\right\}$ according to Liu et al. (2009) as the solution **w** to the formula 6 will decay to zero if they were set ≥1. λ_1_ was set on the logarithmic scale for quick search, while λ_2_ was set on the natural scale with λ_2_ > λ_1_ to incorporate more intergroup sparsity than intragroup sparsity. The similar parameter setting is applied in Zheng et al. (2021), Liu et al. (2022), and Zheng et al. (2018).

**Figure 3:**
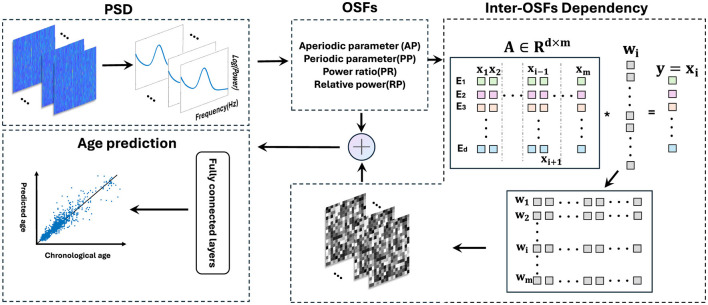
Schematic flowchart of BA prediction. Extract four sets of electrode-wise features from MNCS dataset. Adopting the sparse group lasso, with the vector of one feature as the target variable and the vectors of other features as the regression variables. By repeating this process 13 times, a multi-feature-based ODC matrix was constructed, where each row in the gray matrix reflects a set of regression coefficients from a single sparse regression model. Finally, OSFs and ODC matrix were input into the fully connected layers to predict BA.

The ODC was constructed by the optimal λ. A brief overview of this method is shown in Figure 3. For each subject, this process was repeated *m* times to construct the ODC matrix $\text{W}={\left[{\text{w}}_{1},...,{\text{w}}_{i},...,{\text{w}}_{m}\right]}^{T}\in R^{m\times \left(m-1\right)}$. Each row of **W** represents a set of regression coefficients from a single sparse regression model, with a non-zero value indicating a relatively strong relationship between the target feature vector and the regressor vectors and a zero value indicating a null relationship. Due to each row in the matrix being composed of sparse solutions from different regression processes, the ODC matrix should be asymmetric, representing the dependencies between features.

### 2.7 Prediction model

With the learned OSFs and the ODC, the fully connected neural network (FCNN) is designed for the downstream BA regression task. It includes an input layer, three hidden layers, and an output layer. The input layer receives processed features with a size equal to the number of input features. Each hidden layer contains 10 neurons. The output layer consists of a neuron that outputs the predicted age value. Too many hidden layers may lead to overfitting and a waste of computational resources, while too few hidden layers may not fully capture complex data patterns. The selection of 10 neurons per hidden layer was validated through comparative experiments. Excessive neurons can increase computational burden and lead to overfitting, while insufficient neurons may not fully express the complex internal relationships in the data. In terms of the activation function, the hidden layer adopts the function *tansig*, which maps the input to the range [–1, 1], helping to normalize the output of neurons and stabilize the training process. The output layer adopts a linear activation function *purelin* to ensure the continuity of the output for the regression task. The training process involves forward propagation, backward propagation, and weight updates. In the forward propagation process, the input data passes through each layer, and each neuron applies an activation function to the weighted sum of the input, ultimately producing an output. Backpropagation calculates the error between the output and the target using the Levenberg Marquardt (LM) algorithm (Ranganathan, 2004) to adjust the network weights to minimize the error. The LM algorithm is particularly suitable for training small and medium-sized networks due to its fast convergence. We perform a 10-fold cross-validation by randomly partitioning the entire data set into 10-folds with 90% as the training set and 10% as the test set. Four metrics were used to evaluate prediction performance, namely the mean absolute error (MAE), the coefficient of determination (*R*^2^), the root mean square error (RMSE), the mean absolute percentage error (MAPE) and the respective 95% confidence interval.


(7) $$\begin{array}{l} MAE=\frac{1}{n_{s}}\overset{n_{s}}{\underset{i=1}{\sum }}|y_{i}-{\hat{y}}_{i}| \end{array}$$

(8) $$\begin{array}{l} R^{2}=1-\frac{\sum_{i=1}^{n_{s}}{\left(y_{i}-{\hat{y}}_{i}\right)}^{2}}{\sum_{i=1}^{n_{s}}{\left(y_{i}-\bar{y}\right)}^{2}} \end{array}$$

(9) $$\begin{array}{l} RMSE=\sqrt{\frac{1}{n_{s}}\overset{n_{s}}{\underset{i=1}{\sum }}{\left(y_{i}-{\hat{y}}_{i}\right)}^{2}} \end{array}$$

(10) $$\begin{array}{l} MAPE=\frac{1}{n_{s}}\overset{n_{s}}{\underset{i=1}{\sum }}|\frac{y_{i}-{\hat{y}}_{i}}{y_{i}}|*100\% \end{array}$$

where ${\hat{y}}_{i}$ is the predicted age, *y*_*i*_ is the actual chronological age based on the date of birth, ȳ is the average actual age, and *n* is the total number of samples.

Meanwhile, we applied the layerwise relevance propagation (LRP) (Bach et al., 2015) to examine which input features are important to the output. LRP is an interpretability method for neural networks that utilizes backpropagation technology. Based on the relevance between a neuron and the output decision $R_{k}^{\left(l+1\right)}$, the decomposition of the relevance between the previous layer of neurons, denoted $R_{i\leftarrow k}^{\left(l,l+1\right)}$, is obtained as follows:


(11) $$\begin{array}{l} R_{i\leftarrow k}^{\left(l,l+1\right)}=R_{k}^{\left(l+1\right)}\cdot \frac{a_{i}w_{ik}}{\sum_{i=1}^{n}a_{i}w_{ik}+b_{k}} \end{array}$$

where *a*_*i*_ is the activation value of the neuron with index *i* in the *l*-th layer, *w*_*ik*_ is the weight connecting the neuron *i* in the *l*-th layer and the neuron *k* in the (*l* + 1)^*th*^ layer, and *b*_*k*_ is the bias in the (*l* + 1)^*th*^ layer. The relevance of a certain neuron in the *l*^*th*^ layer is decomposed from the relevance of each neuron in the next layer with the following formula:


(12) $$\begin{array}{l} R_{i}^{\left(l\right)}=\overset{n}{\underset{k=1}{\sum }}R_{i\leftarrow k}^{\left(l,l+1\right)} \end{array}$$

According to Equations 11, 12, the relevance for a certain neuron can be calculated as:


(13) $$\begin{array}{l} R_{i}^{\left(l\right)}=\overset{n}{\underset{k=1}{\sum }}R_{k}^{\left(l+1\right)}\cdot \frac{a_{i}w_{ik}}{\sum_{i=1}^{n}a_{i}w_{ik}+b_{k}} \end{array}$$

To prevent $R_{i\leftarrow k}^{\left(l,l+1\right)}$ from taking an infinite value, this algorithm divides the relevance into positive and negative. Therefore, the final formula for calculating the relevance of a certain neuron is as follows:


(14) $$\begin{array}{l} R_{i}^{\left(l\right)}=\overset{n}{\underset{k=1}{\sum }}R_{k}^{\left(l+1\right)}\left(\frac{{a_{i}w_{ik}}^{+}}{{\sum_{i=1}^{n}a_{i}w_{ik}}^{+}+b_{k}}+\frac{{a_{i}w_{ik}}^{-}}{{\sum_{i=1}^{n}a_{i}w_{ik}}^{-}+b_{k}}\right) \end{array}$$

Here, the relvance for each OSF between the transformed inputs at the first hidden layer and the output decision (BA) was computed. Then the relvance for each group of OSFs was computed by summing the relvances of each OSF that belongs to the specific group.

## 3 Results

### 3.1 Lifespan evolutionary trajectories of OSFs

**Figure 4:**
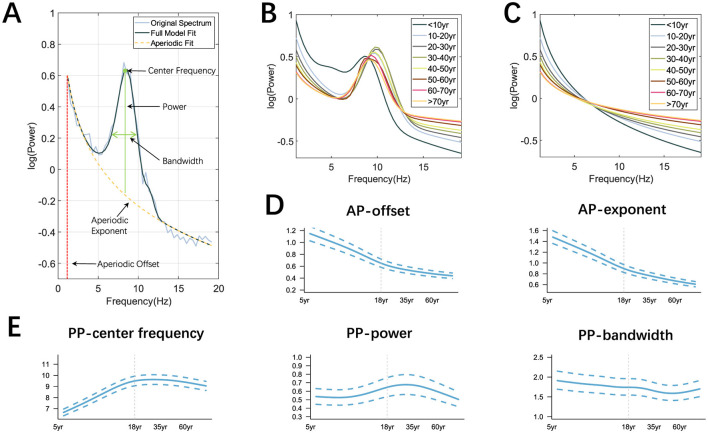
Extraction of OSFs and the trajectories of AP and PP. **(A)** Illustrive fitting with the AP-exponent (in yellow) and AP-offset (in red), as well as the PP-center frequency, PP-power, and PP-bandwidth (in green) on the O1 electrode; **(B, C)** Power spectra and AP for each age group; **(D, E)** Age-depdendent trajectories of AP and PP. Note that age is in the log scale and the shown curves in **(B–E)** were the averaged values over all the electrodes.

Figure 4A is an illustrative process for spectral fitting using FOOOF. Prominent differences in power spectra can be seen between age groups (Figure 4B) and the aperiodic components were shown as Figure 4C. The spectral decomposition model effectively captured the differences between different age groups. The peak of the power spectrum increased with age and reached a maximum at the age of 40 years, after which it gradually decreased. The largest difference in AP was observed between the <10 years group and the 10–20 years group. The AP-exponent and AP-offset (Figure 4D) rapidly decreased from the beginning of life until the age of 18, when they slowly decreased, PP−center frequency (Figure 4E) rapidly increased before the age of 18 and then slowly decreased, while the trend of PP−power and PP−bandwidth changes was not quite significant.

**Figure 5:**
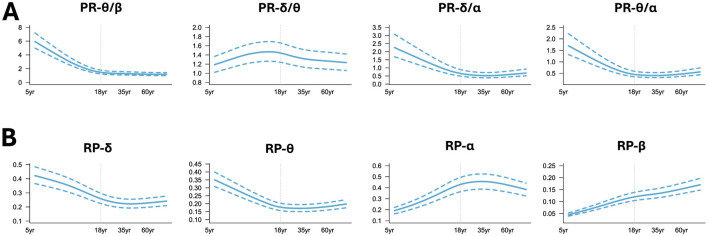
Normative trajectory of band power. **(A)** Power ratio (PR); **(B)** Relative power (RP). Note that all the shown curves were the averaged values over all the electrodes.

Meanwhile, the GAMLSS model was applied to traditional band powers (PR, RP) to capture their age-dependent trajectories. We found that PR-θ/β, PR-δ/α, PR-θ/α (Figure 5A) decreased first with age and tended to plateau after adulthood. PR-δ/θ increased in the early stages of life until the age of 18 years and showed a downward trend after adulthood. In the low-frequency band of RP (Figure 5B), RP-δ and RP-θ decreased with age and slowly increased around adulthood. RP-α increased first with age and slowly decreased around middle age, while RP-β showed an upward trend throughout the lifespan, but the rate of increase varied in different age groups. Note that the curves shown in Figures 4B–E were the averaged values over all electrodes with the aim of mapping the global trajectories across age groups, meaning that the electrode differences were not of key concern, which can be referred to our previous work (Hu et al., 2019a).

### 3.2 PCC analysis between OSFs and BA

**Figure 6:**
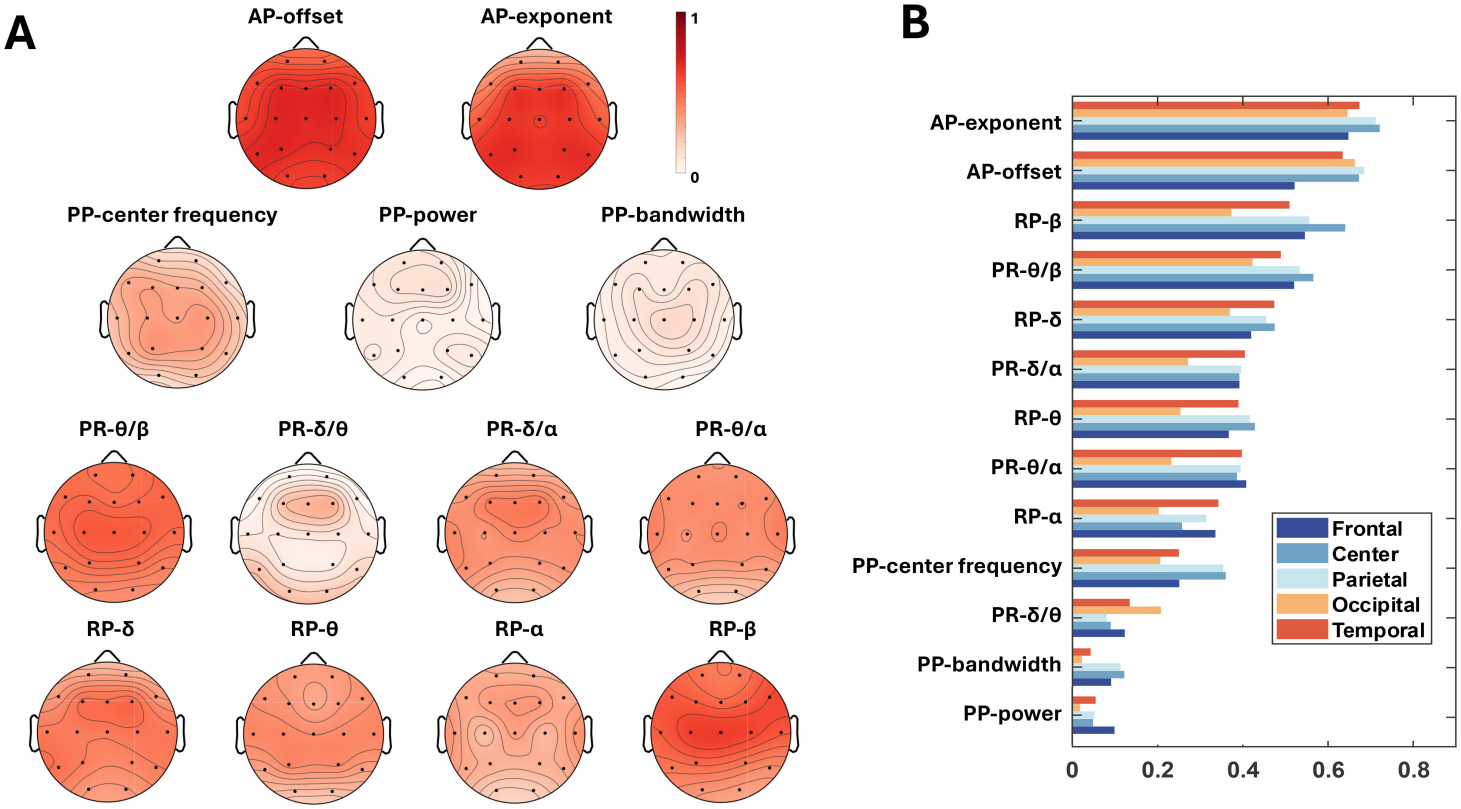
Feature importance for age prediction. **(A)** Topographies of electrode-wise PCCs between each OSF and BA. Electrodes from left to right, from anterior to posterior, are Fp1, Fp2, F7, F3, Fz, F4, F8, T3, C3, C4, T4, T5, P3, Pz, P4, T6, O1, O2; **(B)** The PCCs of OSFs as to brain region.

The PCCs between 13 OSFs with BA were anlyzed in terms of electrodes and brain regions. Figure 6A depicted the scalp topographies of the electrode-wise PCCs between the OSFs and the BA. In Figure 6A, each row represents a group of features, and the topographies of electrode-wise PCCs exhibit a generally symmetric distribution. It may indicate that no difference between the left and right hemispheres should be considered for the prediction of BA when using OSFs. From the perspective of intragroup OSF comparison, the AP exponent, the AP offset, the PP center frequency, PR-θ/β and PR-δ/α, as well as RP-δ and RP-β were found to be more important compared to other OSFs within the respective group. The bar charts in Figure 6B is the PCC between OSFs and BAs across the brain regions, where the OSFs were arranged in descending order. For OSFs such as the AP-exponent, the AP-offset, the PP-center frequency, PR-θ/β and RP-β, the central and parietal regions exhibit the larger PCCs than the other regions, while the lower PCCs are observed in the occipital region. In contrast, PR-δ/θ shows a higher PCC in the temporal and occipital areas compared to other regions. It suggests that the PCCs of the OSFs show slight variations with brain regions. However, seen from the descending order of PCCs in the Figure 6B, the variations in the PCCs as to the group and type of OSF are greater than those of the hemisphere and the brain region. This implies that the selection of OSFs is necessary for the prediction of BA.

### 3.3 Performances of BA prediction models

Each group of fused OSFs was entered into the prediction model to evaluate the prediction performances of that group. We applied the traditional band power, PR and RP, as the first two important baselines. The prediction performances are shown in Table 1. Evidently, it showed that the traditional band powers offered the worse performances than AP in all four metrics of MAE, *R*^2^, RMSE, and MAPE. With intergroup comparison among PR, RP, PP, AP, it is easily found that the best prediction result is obtained with AP, with a MAE of 3.69 years and *R*^2^ of 0.83. When all OSFs were fused as input, MAE decreased to 3.44 years and *R*^2^ increased to 0.84. In contrast to OSFs, the MAE increased slightly to 3.71 and the RMSE increased to 5.34 if the ODC was input only. This showed that ODC was worse than OSF and AP, perhaps due to the lack of neurophysiological interpretability and sensitivity. With both ODC and OSF concatenated as model input, MAE decreased to 2.95 and *R*^2^ increased to 0.86, with $\lambda_{1}=2^{-6}$ and λ_2_ = 0.1 as shown in Table 2 reflecting how MAE varied with the values of λ. From Table 2, it can be summarized that MAE tends to be smaller and less sensitive to the variation of λ if $\lambda_{1}\in \left[2^{-6},2^{-9}\right]$ and λ_2_ ∈ [0.1, 0.3] are compared to λ which fall into other regions.

**Table 1:** Feature comparison with PR, RP, PP as baselines [confidence intervals].

**Feature**	**MAE**	***R* ^2^**	**RMSE**	**MAPE (%)**
PR	4.40 [4.01, 4.79]	0.78 [0.75, 0.81]	6.30 [5.82, 6.78]	18.21 [16.86,19.56]
RP	5.21 [4.56, 5.86]	0.72 [0.67, 0.76]	7.38 [6.68, 8.09]	22.73 [21.52,23.95]
PP	8.38 [8.05, 8.71]	0.51 [0.48, 0.54]	11.95 [11.39, 12.51]	38.54 [36.29,40.79]
AP	3.69 [3.41, 3.96]	0.83 [0.81, 0.85]	5.42 [4.98, 5.87]	15.23 [14.51,15.96]
OSFs	3.44 [3.07, 3.81]	0.84 [0.82, 0.86]	5.08 [4.60, 5.57]	15.43 [14.44,16.42]
ODC	3.71 [3.25, 4.17]	0.84 [0.81, 0.87]	5.34 [4.75, 5.92]	15.07 [14.04, 16.09]
**OSFs-ODC**	**2.95 [2.66, 3.24]**	**0.86 [0.82, 0.91]**	**4.51 [3.88, 5.13]**	**12.44 [10.90, 13.98]**

The values in bold are the results of our proposed upstream method.

**Table 2:** Sensitivity analysis of the (λ_1_, λ_2_) selection.

**λ_1_, λ_2_**	**0**	**0.1**	**0.2**	**0.3**	**0.4**	**0.5**	**0.6**	**0.7**	**0.8**	**0.9**
**2** ^**-1**^	3.54	3.58	4.06	4.35	4.27	3.32	3.83	4.13	4.25	3.96
**2** ^**-2**^	3.14	3.81	4.20	3.98	3.34	4.08	4.49	4.75	5.36	3.84
**2** ^**-3**^	4.31	3.74	3.08	3.93	4.21	4.54	3.36	3.03	3.40	4.00
**2** ^**-4**^	4.52	6.44	4.85	3.13	4.13	3.39	5.50	5.61	3.44	3.50
**2** ^**-5**^	3.31	4.27	3.27	5.41	4.57	3.73	3.48	3.92	3.13	4.26
**2** ^**-6**^	3.24	**2.95**	3.31	3.66	3.34	3.35	3.79	3.69	4.69	3.13
**2** ^**-7**^	3.61	3.61	3.14	2.99	3.89	4.24	3.09	4.08	3.38	3.12
**2** ^**-8**^	3.33	3.23	3.69	3.35	3.94	3.74	5.17	3.79	3.28	3.15
**2** ^**-9**^	3.39	3.23	3.96	3.09	4.26	3.12	4.13	3.14	5.23	3.91
**2** ^**-10**^	2.99	3.29	4.27	4.20	4.92	3.40	3.08	3.01	4.07	4.80

The value in bold is the least MAE.

To validate the effectiveness of the downstream prediction model, we systematically conducted baseline comparisons, comprehensively comparing FCNN with baseline methods such as linear regression (LR), support vector regression (SVR), ridge regression (RR), and random forest (RF). As shown in Table 3, the experimental results show that the MAE followed the order FCNN (2.95) < RF (4.51) < LR (5.04) < SVR (5.47) < RR (8.90). Thus, FCNN showed 34.6% advances compared to RF, and sensibly outperformed the other baseline methods. Later, it was combined with the LRP to significantly improve the interpretability of the model. Our proposed full model is “OSFs-ODC-FCNN” for neural oscillation-based brain age (NEOBA) prediction, where “OSFs-ODC” is in the upstream of NEOBA for feature construction and the FCNN is in the downstream of the NEOBA model for age prediction. To verify the effect of NEOBA on the independent dataset, we implemented validation in the TDBRAIN dataset, which is an open clinical EEG database with 1,274 subjects aged 5–89 years (Khayretdinova et al., 2022). The TDBRAIN dataset was analyzed using the three conditions: eyes open and closed, eyes open only, and eyes closed only, with the MAE results shown in Table 4. When using the end-to-end deep convolutional neural network (DCNN) model proposed in Khayretdinova et al. (2022), MAE (years) were 5.96, 6.39, and 6.33, respectively. With our proposed “NEOBA” model, the MAE was reduced to 4.95, 5.62, and 5.24, with improvements of 16.94%, 12.05%, and 17.21%, respectively.

**Table 3:** Comparison with basic regression methods.

**Methods**	**MAE**	***R* ^2^**	**RMSE**	**MAPE (*%*)**
LR	5.04	0.79	7.18	24.63
SVR	5.47	0.84	7.43	23.02
RR	8.90	0.69	11.96	39.17
RF	4.51	0.85	6.69	16.83
**FCNN**	**2.95**	**0.86**	**4.51**	**12.44**

The values in bold are the results of our proposed downstream method.

**Table 4:** Comparative experiment on TDBRAIN dataset (MAE).

**Methods**	**Eyes states**		
	**Eyes open-closed**	**Eyes open**	**Eyes closed**
DCNN	5.96	6.39	6.33
**NEOBA**	**4.95**	**5.62**	**5.24**

The values in bold are the results of our proposed model.

**Figure 7:**
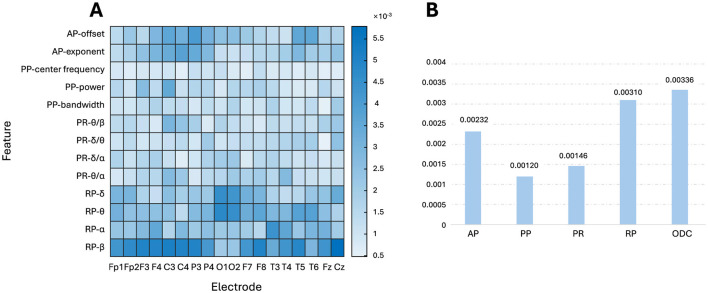
FCNN interpretability by LRP analysis. **(A)** Relevancies between the transformed OSFs and the output BA; **(B)** relevancies between the group of OSFs or ODC and the output BA.

To gain explcit model interpretability, LRP analysis was performed to calculate the relevance between the first hidden layer of FCNN and the output layer by backpropagating. The first hidden layer was analyzed because it is responsible for learning the first transformations between the input and the later predictions. Figure 7A shows the relevances corresponding to each OSF per electrode. Figure 7B shows the relevance of each group of OSF by averaging on intragroup OSFs and electrodes and the relevance of ODC by averaging all entries within the ODC matric, further quantifying their importance in the age prediction task. Note that the sum of all the relevances in Figure 7B is 1. The relevance values in Figure 7 AB are small, because the flatten feature dimension is as large as 390 × 1 from the size 18 electrodes × 13 OSF (two APs, four PRs, four RPs, and three PPs) and the size 13 × 12 for the ODC matrix. As seen in Figure 7B, ODC occupied a pivotal position in the age prediction process, closely followed by RP and AP. Delving deeper into the Figure 7A, it was found that RP-β maintained a high importance in most brain regions, except the occipital regions compared to RPs in the other bands, while RP-α showed a more balanced spatial distribution throughout the brain. The relevances of the RP-δ and RP-θ bands were higher in the occipital regions, showing the sensitivity of this specific region to RP in these bands. The relevances were higher in the central and parietal regions of the AP.

Moreover, the NEOBA model was validated within the specific age range. The four age ranges are adolescents aged 5–18, youth aged 18–45, middle aged 45–60, and elderly aged >60. As shown in Table 5, the minors achieved the best MAE of 2.33 and *R*^2^ of 0.68. The youth group had an MAE of 2.83 and an *R*^2^ of 0.61. In contrast, the middle-aged group and the elderly group had much larger age prediction errors with MAE of 5.03 and 4.73 and *R*^2^ of 0.34 and 0.25, respectively.

**Table 5:** Performance under different age groups.

**Age**	**Sample size**	**MAE**	***R* ^2^**	**RMSE**	**MAPE (%)**
5–18	485	2.33	0.68	2.86	24.86
19–45	881	2.83	0.61	4.33	11.34
46–60	53	5.03	0.34	7.44	9.49
61–97	208	4.73	0.25	7.24	6.51
**5–97**	**1,628**	**2.95**	**0.86**	**4.51**	**12.44**

The values in bold are the results of our proposed lifespan age prediction.

## 4 Discussion

In this study, we first applied FOOOF to extract OSFs and then mapped the age-dependent trajectory of OSFs using GAMLSS. Evident differences in terms of power spectrum and AP were found between the age groups. The offset of AP and the exponent of AP was observed to decrease rapidly in the early stages of the lifespan, while the α PP-center frequency increased rapidly before adulthood. These results reveal the evolutionary patterns that brain functions may experience at different stages of life. In addition, we show that AP and PP play a key role in predicting BA, especially in the central and parietal regions. The significance of these OSFs in specific brain regions should be linked to the pronounced neurofunctional reorganization associated with normal development and aging processes. Secondly, the constructed ODC matrix integrated the interaction information among the 13 OSFs by applying the sparse group lasso and demonstrated its superiority over the most OSFs in predicting BA. Comparison experiments with baselines indicated that ODC in combination with OSFs can greatly improve the prediction performances; and the FCNN outperformed all basic regression models tested. Lastly, the application of LRP analysis to FCNN uncovered the different contributions of OSFs and ODCs in age prediction that were ODC > RP > AP > PR > PP. This work established a novel analytical framework, NEOBA, that efficiently integrates lifespan-related neural oscillations for accurate prediction of BA, while offering crucial insights into the neural mechanisms underlying developmental trajectories and senescence processes.

The two APs, offset and exponent, decreased rapidly in the early stages and then slowly in adulthood, as shown in Figure 4D. Compared to young people, older people exhibited a flatter power spectrum as shown in Figure 4C, which is consistent with (Voytek et al., 2015; Tran et al., 2020). Similar patterns have also emerged when considering both adults and children, with exponent and offset negatively correlated with age (He et al., 2019), and adults have higher center frequencies of the α peak than children as shown in Figure 4E, indicating higher cortical maturity (Marshall et al., 2002). In general, the above results are in line with other neurophysiological findings that exponent and offset are reliable markers that exhibit nonlinear decline throughout the development process and may lead to age-related changes in cognitive function (Tran et al., 2020).

The offset and exponent of AP are closely related to brain development and psychopathology (Molina et al., 2020; Tran et al., 2020). Gao et al. (2017) suggests that AP may indicate the balance between excitation and inhibition in cortical circuits. The lower exponent reflects the broadband flattening of PSD, indicating transfer from cortical inhibition, whereas the higher exponent indicates opposite activation patterns. It also provides insight into the physiological mechanisms underlying information interruptions in neurodevelopmental disorders (Ostlund et al., 2021; Robertson et al., 2019), autism (Levin et al., 2020), and schizophrenia (Molina et al., 2020). The AP offset showed fair test-retest reliability in typical developmental and autistic children (Levin et al., 2020). For example, children with ADHD have a larger offset compared to healthy peers (Robertson et al., 2019). Levin et al. (2020) demonstrated the utility of the spectral shape and other parameters as stable biomarkers of cortical activity and disease diagnosis. The clear advantages of incorporating the AP into the EEG-based BA studies can be inferred from existing studies that spectral parameterization can help with the appropriate physiological interpretation by accurately decomposing the spectral components. It confirms that traditional spectral features such as PP are no longer sufficient, as they may be conflated by aperiodic components (Donoghue et al., 2020a). The joint use of AP and traditional band powers verified the consistency of these two components, providing a solid reference for building accurate and reliable age prediction models.

Note that in low signal-to-noise ratio scenarios, FOOOF may not accurately estimate oscillation parameters such as center frequency, power, and bandwidth, as well as AP such as exponent and offset (Donoghue et al., 2020b). Furthermore, the differences in EEG features across age groups can lead to biased parameter estimation. Children EEG often have higher noise levels, while elderly EEG may show flatter power spectra and lower power peak (Schaworonkow and Voytek, 2021; Voytek et al., 2015). These factors may influence the accuracy and reliability of the research findings. Thus, it is recommended to combine strict data preprocessing and feature validation methods when applying ξπ to minimize their potential impact (Hu et al., 2024). In addition, the EEG OSFs did not cover all age-related EEG biomarkers. Other EEG analysis, such as functional connectivity and microstates (Hu et al., 2018a), may provide additional information to provide a comprehensive understanding of BA changes.

The construction of the interaction matrix between multiple features based on sparsity has been widely used in the medical field for the diagnosis of diseases (Zheng et al., 2021; Liu et al., 2022; Zheng et al., 2018). Here, the sparse group lasso was applied to construct the ODC matrix because of its ability to handle both intergroup and intragroup sparsity simultaneously. In contrast, the Lasso with *L*_1_ regularization picks individual OSF while ignoring the inherent group structure (Zheng et al., 2021); and the Elastic net addresses collinearity issues but lacks an explicit group-wise selection mechanism (Xu et al., 2023). By imposing the group sparsity, the ODC matrix achieves two goals: (1) the intergroup sparsity can identify entire feature groups that contribute little to the prediction task; (2) the intragroup sparsity further can identify the most predictive features from the preserved groups with biologically meaningful interaction patterns between these groups. Our findings indicate that the incorporation of ODC in the upstream input reduced the MAE by 14.24% compared to using only OSFs. Both OSFs and ODC may be complementary and together provide a comprehensive description of neural oscillation. This underscores the necessity of including ODC in model input, a point not addressed in previous studies. Meanwhile, we observed that the prediction results are age related, and older participants have poorer prediction results, which may reflect the large individual variation due to brain atrophy (Amoroso et al., 2019; Kumari and Sundarrajan, 2024).

The feature selections generally include filtering methods based on the correlation and the information score, the wrapper search methods, such as recursive elimination, stepwise selection, and the embedded methods in the model training process to avoid overfitting. Common embedded methods with regularization are the lasso (*L*_1_ norm), the ridge regression (*L*_2_ norm), the elastic net (*L*_1_ + *L*_2_ norm) and the group lasso (*L*_2_ norm with group) and the sparse group lasso (*L*_1_ + *L*_2_ norm with group). The lasso and elastic net enable the selection of individual features, while the elastic net is more stable than the lasso by alleviating collinearity using the *L*_2_ norm. However, the lasso, the ridge regression, and the elastic net were unable to perform group selection. Unlike a group lasso that may eliminate the whole group, the sparse group lasso enables both inter-group and intra-group selection, simultaneously. In this work, we predefined the four physiologically meaningful groups of OSFs, such as AP, PR, RP, and PP according to quantitative spectra analysis. Since the number of intragroup features is not large, we intended to retain as more intergroup OSFs as possible, thus making it necessary to apply the sparse group lasso. The ODC matrix is the interactive representation among all OSFs, uncovering crucial information hidden in the OSFs.

Figure 6A shows that the PCCs are not uniform for different electrodes or different OSFs, indicating that key factors in predicting BA should not be uniquely attributed to a specific channel or OSF. Each toporaphy is nearly symmetric between the left and right hemispheres, but the PCCs are significantly different over brain regions as shown in Figure 6B. This suggests that the fusion of multiple OSFs from all electrodes can improve the accuracy of the BA prediction. Both PCC and LRP comprehensively evaluate the role of EEG OSFs in age prediction (Lee Rodgers and Nicewander, 1988; Bach et al., 2015). PCC provides a statistical perspective to analyze how upstream OSFs correlated with BA. LRP further reveals the specific contributions of the FCNN input, such as the roles of RP-β in most regions and AP in the central region. The LRP analysis shown in Figure 7 provided an additional perspective to evaluate how the upstream features were weighted during the downstream FCNN transformation. As FCNN acted as the black box, LRP gained transparency on how FCNN processed the input through weighting and updating in the hidden layer. The LRP revealed that the contribution of different features for the prediction of BA followed the order: ODC > RP > AP > PR > PP, which generally followed the feature importance as shown in Figure 6. The LRP result provided direct evidence to interpret why the addition of ODC could improve the accuracy of the BA prediction as shown in Table 2.

Researchers use multiple modalities and methods to explore the patterns and mechanisms of brain aging (Rutledge et al., 2022). Functional magnetic resonance imaging (fMRI) is a common modality that has significant advantages in capturing functional changes as well as anatomical changes. For example, Chang et al. (2024) used the resting fMRI and LASSO to identify the 39 features that are most relevant to BA, established a prediction model, and found that the default mode network is associated with abnormal aging, adding insights to BA prediction and biomarker research. EEG can effectively capture the dynamic changes of neural oscillations due to high temporal resolution. Various models have been applied to predict BA, such as support vector machines, ensemble learning, random forests, correlation vector machines, and deep convolutional neural networks (DCNN), to predict BA (Dimitriadis and Salis, 2017; Al Zoubi et al., 2018; Vandenbosch et al., 2019; Khayretdinova et al., 2022). Sun et al. (2019) adopted the temporal statistical indicators and band powers, PR and RP, as upstream features and an interpretable machne learning model for the prediction of BA based on sleep EEG. They reported that the EEG time-frequency features were superior to the sleep macrostructure features in the BA prediction. Their results showed a 7.6-year MAE in age prediction of 1,022 healthy participants using only six electrodes and demonstrated a stable increase in BA over time in longitudinal validation. Furthermore, participants with neurological or mental diseases showed an average BA index of 4 years older than the healthy group, while patients with hypertension and diabetes showed an average BA of 3.5 years older, indicating that sleep EEG may be an effective modality for the prediction of BA. Currently, there are few studies in the field of brain age prediction using scalp resting EEG, and high-quality resting EEG datasets with the actual age values included are scarcely available. This limits the possibility of fully and objectively comparing the performances of different prediction models on diversified datasets and thus makes it difficult to comprehensively assess the generalization ability and robustness of each model. In particular, the MNCS dataset in this study is not the raw EEG time series but the cross-spectra, thus limiting the direct application of other methods to this dataset. To date, only Jarne et al. (2024) has attempted the BA prediction using the MNCS dataset, and their model yielded results with an MAE of about 10 years and an *R*^2^ of about 0.55. Compared to DCNN (Khayretdinova et al., 2022), our NEOBA model has improved by reducing the MAR by 16.95% in the TDBRAIN dataset. NEOBA demonstrates significant superiority on both the MNCS dataset and the TDBRAIN dataset.

It should be noted that the MNCS data set may be affected by possible confounders, such as multisite data acquisition, variations in electrode placement, and harmonization strategies. The details of the MNCS data set can be accessed in Table C1, Appendix C of our previous work (Li et al., 2022b). Because it is a data set coshared by the call for international collaboration by the Global Brain Consortium, there are significant differences in the recording protocols for the different batches. Two steps were implemented to homogenize the differences across all sites (counties), devices and years of recordings, as detailed in Table D1 of Li et al. (2022b). The first step was to extract the electrodes with the standard 10–20 electrode placement system (Hu et al., 2018b), to restrict the frequency range to 1.17–19.14 Hz to include the legacy data set of 211 subjects from Cuba (Bosch-Bayard et al., 2020), and to provide unified instruction for the cleaning of artifacts and the standardized all-in-one script for cross-spectral calculation. The second step was to investigate the impacts of batches, that was defined as the sites, devices, and the years (studies) of recordings.

It is interesting to discover gender-dependent changes as BA is expected to be older in women during puberty and younger in female during adulthood. The reason why sex-dependent changes were not considered is as follows. (1) The MNCS data set contains 908 males, 912 females, and 146 unknown cases. The age distribution for either gender will be less balanced with reduced sample count, preventing the normative study for the lifespan. It has been shown that “gender” has no dependent effect on normative modeling applying a mixed-effects model, while “age” and “frequency” are crucial (Li et al., 2022b; Hu et al., 2019a). However, this issue deserves to be addressed in the future if the data size gets much larger. This will enable to better understand how gender affects BA prediction.

In the future, the spatiotemporal and spatiospectral features of the EEG can be extracted to systematically evaluate the importance of different types of features and identify the features that best reflect brain aging. Longitudinal designs can capture true individual changes over time, which is crucial for understanding dynamic brain structure and function (Bethlehem et al., 2022). Longitudinal data can validate and improve models built from cross-sectional data, enhancing model generalization and stability. In addition, longitudinal designs support early intervention by detecting abnormalities early, facilitating early diagnosis and disease progression. Since longitudinal data are difficult to obtain, most lifespan EEG studies are based on cross-sectional data (Jockwitz and Caspers, 2021; Rosenberg et al., 2020; Wang et al., 2012). To ensure the stability and reliability of the model, future research should prioritize longitudinal studies. At the application level, based on EEG features, BA prediction models are used as auxiliary diagnostic tools for the early detection of neurodegenerative diseases or other age-related health problems. Automated prediction of brain age from raw EEG is theoretically feasible, as it only requires additional pre-processing and spectral calculation steps. All the preprocessing, the spectral calculation, and the downstream prediction can be integrated into a complete standalone pipeline. If large normative EEG time series data are available with brain age values for use, fully automated end-to-end deep learning approaches can be developed with the latest advances in time series state-space modeling, Fourier neural network, and generative models.

## 5 Conclusion

This study constructed for the first time the evolutionary trajectory of EEG OSFs over the lifespan and proposed a novel framework named NEOBA, i.e., OSFs-ODC-FCNN, to predict BA. The ODC matrix was learned by the sparse group lasso, reflecting interactive representational information across multiple OSFs. Integrating ODC with OSFs achieved optimal performance in MNCS and TDBRAIN datasets. Through PCC and LRP, the role of OSFs and FCNN in age prediction tasks was systemically evaluated, gaining us more interpretability and reliability. It indicates that the joint input of both ODC and OSFs can greatly improve the prediction of BA. The code and tutorial of the NEOBA framework are freely available at https://github.com/ShiangHu/NEOBA.

## Data Availability

Publicly available datasets were analyzed in this study. This data can be found at: https://doi.org/10.7303/syn26712693.
